# The effect of empagliflozin on renal outcomes compared to sitagliptin with type 2 diabetes: target trial emulation using electronic medical records

**DOI:** 10.1007/s11096-025-02024-9

**Published:** 2025-10-23

**Authors:** Min Ju Kang, Hyun Kyung Lee, Minoh Ko, Ha Young Jang, In-Wha Kim, Jung Mi Oh

**Affiliations:** 1 College of Pharmacy, Seoul National University, 1, Gwanak‑Ro, Gwanak‑Gu, Seoul 08826 Republic of Korea; 2 College of Pharmacy, Daegu Catholic University, Gyeongsan, 38430 Republic of Korea; 3 College of Pharmacy, Gachon University, Incheon, 21936 Republic of Korea; 4 Research Institute of Pharmaceutical Sciences, Seoul National University, Seoul, 08826 Republic of Korea; 5 Natural Products Research Institute, Seoul National University, Seoul, 08826 Republic of Korea

**Keywords:** Chronic renal insufficiency, Dipeptidyl-peptidase IV inhibitors, Sodium-glucose transporter 2 inhibitors, Type 2 diabetes mellitus

## Abstract

**Introduction:**

The renal effects of sodium-glucose cotransporter 2 (SGLT2) inhibitors in diverse populations remain under investigation. While randomized controlled trials have shown renoprotective effects of SGLT2 inhibitors, their impact in routine clinical settings and specific populations such as Koreans requires further evaluation.

**Aim:**

This study aimed to evaluate the impact of empagliflozin compared to sitagliptin on renal function and related clinical outcomes in patients with type 2 diabetes (T2D) using a target trial emulation approach.

**Method:**

We conducted a retrospective cohort study using electronic medical records from a Korean tertiary care hospital between 2018 and 2019. New users of empagliflozin or sitagliptin were identified and matched using propensity scores to control for confounding factors. Primary outcomes included changes in estimated glomerular filtration rate (eGFR) and other renal function markers over time. Secondary outcomes included acute kidney injury, albuminuria, and composite renal events, as well as other clinical parameters. A modified intention-to-treat analysis was performed, with follow-up up to 13 months.

**Results:**

After matching, 219 T2D patients were identified in each group. Empagliflozin was associated with a slower decline in eGFR compared to sitagliptin (*p* < 0.05). Serum phosphorus levels increased more with empagliflozin (*p* < 0.05). Empagliflozin showed a non-significant trend toward lower risk of composite renal outcomes (hazard ratio [HR] 0.78; 95% confidence interval [CI], 0.50–1.22). Significant increases in weight loss (HR 2.95; 95% CI, 2.01–4.33) and urination frequency (HR 4.05; 95% CI, 1.14–14.34) were observed with empagliflozin. Serum uric acid levels decreased more in the empagliflozin group (*p* < 0.05).

**Conclusion:**

This real-world study suggests that empagliflozin may offer renoprotective benefits compared to sitagliptin in T2D patients. However, the increased serum phosphorus levels warrant careful monitoring. These findings provide valuable insights for clinical decision-making in managing T2D patients at risk of renal complications.

**Supplementary Information:**

The online version contains supplementary material available at 10.1007/s11096-025-02024-9.

## Impact statements

- This study demonstrates that empagliflozin use is associated with a slower decline in renal function compared to sitagliptin in Korean patients with type 2 diabetes in a real-world setting.
- By emulating a target trial using EMR data, our findings support the broader applicability of the renoprotective effects of empagliflozin beyond high-risk cardiovascular populations.
- The results suggest that empagliflozin may be a preferred therapeutic option compared to sitagliptin for type 2 diabetes patients at risk of renal complications.
- Additionally, the study highlights the need for routine monitoring of serum phosphorus levels during long-term use, providing practical insights for safer prescribing and patient management in clinical pharmacy practice.

## Introduction

Type 2 diabetes (T2D) is a significant risk factor for chronic kidney disease (CKD), with diabetic nephropathy being a leading cause of end-stage kidney disease globally[1]. Recent clinical trials have demonstrated the renoprotective effects of sodium-glucose cotransporter 2 (SGLT2) inhibitors in patients with T2D[2]. SGLT2 inhibitors are antidiabetic agents that lower blood sugar by increasing urinary glucose excretion[3]. Empagliflozin, the first SGLT2 inhibitor shown to protect against cardiovascular diseases, is also poised to significantly benefit kidney function[4]. Although empagliflozin has proven effects in diabetes patients with CKD and cardiovascular diseases[4], it has been also observed that empagliflozin can increase serum creatinine and decrease estimated glomerular filtration rate (eGFR), which was particularly pronounced in patients with moderate renal impairment[5]. However, its renal effects compared to other antidiabetic medications, such as dipeptidyl peptidase-4 (DPP-4) inhibitors, remain underexplored in real-world Asian populations. DPP-4 inhibitors have often been selected as comparators in studies evaluating the outcomes of SGLT2 inhibitors[6–11], as they are commonly prescribed as second-line therapies after metformin. In Korea, sitagliptin was the first DPP-4 inhibitor to be widely used, and the prescriptions of both DPP-4 and SGLT2 inhibitors have been increasing in recent years[12]. Empagliflozin was approved in Korea in 2016, and its clinical use has steadily increased since then. In post-marketing surveillance, cases of acute kidney injury (AKI) requiring hospitalization or dialysis were reported and reflected in the United States Food and Drug Administration (FDA) package insert[5]. Similarly, a post-marketing surveillance study of empagliflozin in Korea reported rare occurrences (0.01–0.1%) of AKI and CKD[13]. Considering the mechanism of action, empagliflozin carries both the risk of kidney injury and the potential for renal protection. Therefore, research on the continuous changes in various kidney-related clinical data associated with the use of empagliflozin is necessary.

Moreover, the generalizability of these findings to diverse populations and real-world settings remains an important area of investigation. Pharmacoepidemiologic studies using real world data can be used to overcome the limits of randomized clinical trials (RCTs). Although several RCTs have identified renoprotective effect of empagliflozin, their settings were strict. Therefore, assessing its effectiveness in real world settings is essential for generalizing findings to clinical applications[14]. In this perspective, numerous studies using real-world data have been conducted. For instance, the renoprotective effectiveness of empagliflozin has been replicated across various healthcare system settings, including studies using medical records, claims, and national registries in the United States (US)[6] and other countries across Europe[7] and Asia[8, 9, 15–17]. Furthermore, the usage of empagliflozin in a specific population, such as non-diabetic patients with CKD, has also been investigated[18]. Similarly, a reduction in the incidence of end-stage kidney disease was confirmed with the use of SGLT2 inhibitors based on claims data in Korea[10]. However, Korean studies have lacked comprehensive renal outcomes, limiting their relevance to clinical decision-making. Therefore, a comprehensive study including various kidney-related outcomes in Korea would provide multifaceted evidence necessary to consider when using empagliflozin.

Target trial emulation (TTE) has emerged as a robust methodological approach in observational studies, designed to minimize biases such as selection bias, immortal time bias, and confounding, which often compromise the validity of traditional epidemiological research[19]. By constructing and emulating hypothetical trials using real world data, TTE offers a sophisticated framework that produces more reliable and clinically relevant research findings. Empagliflozin, recognized for its potential benefits on cardiovascular and renal outcomes, presents an ideal case for employing TTE due to the complex interplay of beneficial and adverse effects observed in clinical and post-marketing studies. The contrasting findings from RCTs and real-world evidence on its impact on renal function, from protective effects to risks of acute kidney injury, underscore the need for meticulous evaluation using robust epidemiological methods. Although TTE has been applied in studies of empagliflozin in multiple countries, its application in Korea remains underexplored[11].

### Aim

This study aimed to evaluate the impact of empagliflozin compared to sitagliptin on renal function in Korean patients with T2D without end-stage kidney disease, using a TTE approach with real-world data. This methodology allows us to address the limitations of previous observational studies and provide more robust evidence for clinical practice.

## Method

### Study design and data source

**Fig. 1:**
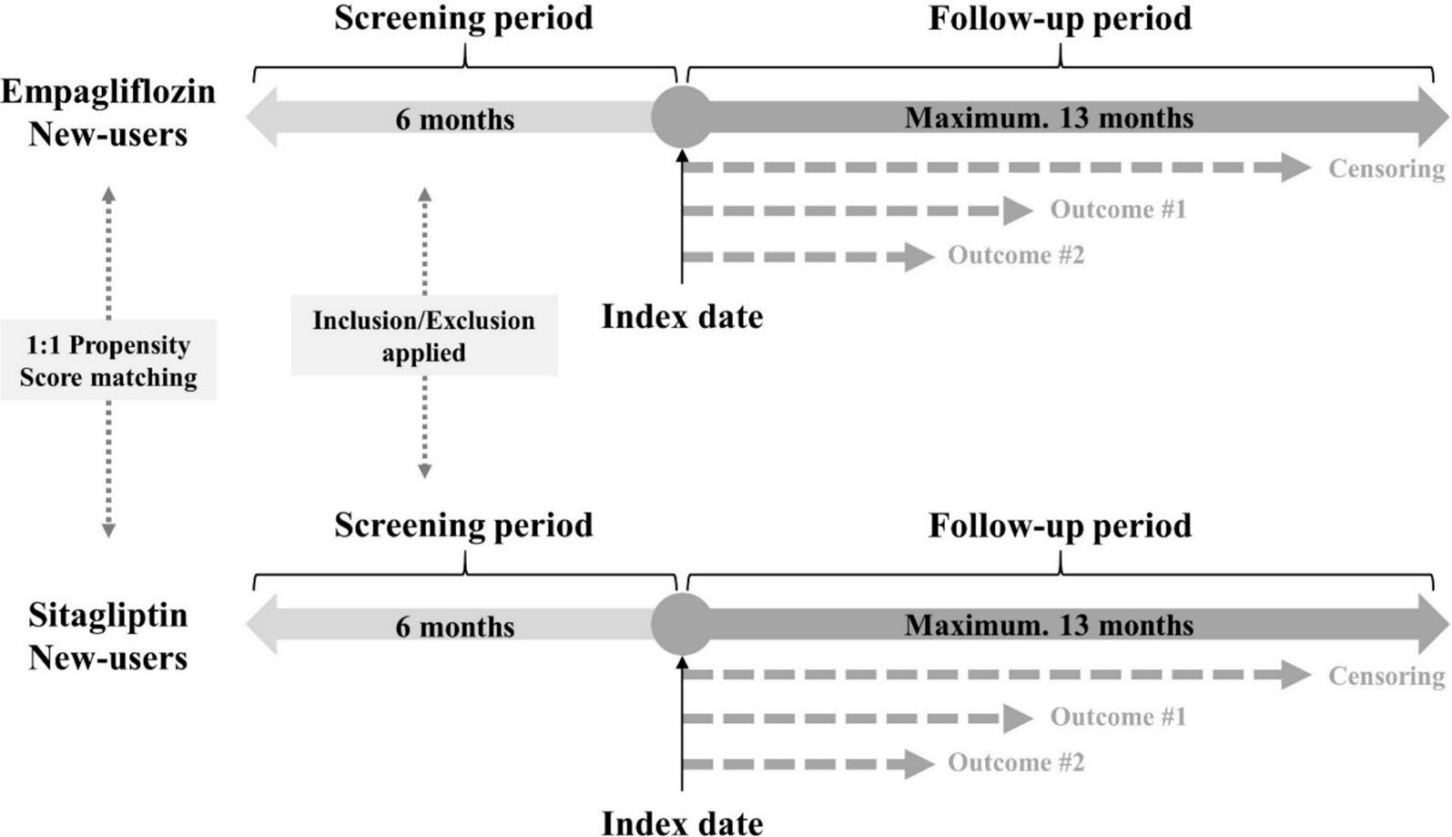
Graphic summary of the study design

This retrospective cohort study followed a TTE framework and used a new-user design with an active control group (Fig. 1). This method minimized biases in observational studies, such as immortal time and selection biases. Eligibility criteria, treatment strategies, and follow-up procedures were carefully defined to mimic a randomized trial. A hypothetical target trial was emulated to assess the association between empagliflozin and its effect on renal function (Table 1). Data were obtained from electronic medical records (EMR) and a clinical data warehouse of a Korean tertiary hospital.

**Table 1:** Protocol of a hypothetical target trial used in the study

Protocol components	Description
Eligibility criteria	Patents with type 2 diabetes mellitus between July 2018 and December 2019, and with no history of taking the same class drug with assigned drug in the past 6 months
Treatment strategies	Empagliflozin group: Initiate empagliflozin at baseline and remain on it during the follow-up unless the patients experience adverse events related to the drug Sitagliptin group: Initiate sitagliptin at baseline and remain on it during the follow-up unless the patients experience adverse events related to the drug
Assignment procedures	Participants will be randomly assigned to either strategy at baseline and will not be aware of the strategy to which they have been assigned
Follow-up	Follow-up starts at randomization and ends at the detection of outcome, death, loss to follow-up, any change in medications of the SGLT2 inhibitors or DPP4 inhibitors, or 13 months after enrollment date, whichever occurs first
Outcomes	Primary outcomes: Composite kidney adverse outcomes (acute kidney injury, albuminuria/proteinuria, end-stage kidney disease, eGFR decline more than 30% from baseline) Secondary outcomes: Acute kidney injury; albuminuria/proteinuria; end-stage kidney disease; eGFR decline more than 30% from baseline; weight loss; increased urination; reduction of antihypertensive medications; change in laboratory levels from baseline on eGFR, systolic blood pressure, diastolic blood pressure, hematocrit, serum sodium, serum potassium, serum chloride, serum calcium, serum phosphorus, and serum uric acid
Causal contrast of interest	Modified intention-to-treat effect
Statistical methods	Modified intention-to-treat effect estimated via 1-year kidney outcomes among individuals assigned to each treatment strategy

SGLT2i, sodium-glucose cotransporter 2 inhibitors; DPP4, dipeptidyl peptidase 4 inhibitors; eGFR, estimated glomerular filtration rate

### Study population

Patients with a diagnosis of T2D (International Classification of Diseases, 10th version [ICD-10] codes: E11–E14) from 1 July 2018 to 31 December 2019 at tertiary hospital and new-users of empagliflozin or sitagliptin were included. New-users were defined as patients who were prescribed a drug and had a washout period of the drug for at least 6 months before the index date of the drug, considering prescription refill gaps. To minimize the possibility of unrecorded prescriptions from other clinics, we reviewed both prescription and medical records, and excluded patients with discrepancies between these records or with less than 6 months of continuous medical records, which could limit adequate assessment. The index date was defined as the prescription date for new initiation of empagliflozin or sitagliptin. Patients were assigned to the first prescribed drug group when eligible as new-users of both empagliflozin and sitagliptin. Patients were excluded if they met any of the following criteria: age < 18 years, no diagnosis of T2D within 6 months before the index date, diagnosis of type 1 diabetes or diabetic ketoacidosis before the index date, eGFR < 30 mL/min/1.73m^2^, diagnosis of end-stage kidney disease except kidney transplantation status, on dialysis at the index date, pregnant at the index date, less than 6 months coverage in EMR, data inconsistencies, no baseline laboratory data, history of using a drug of the same class with assigned drug within 6 months prior to the index date, and concomitant prescription of both SGLT2 inhibitors and DPP4 inhibitors on the index date. The SGLT2 inhibitors and DPP4 inhibitors in this study were restricted to the drugs marketed in Korea (Table S1).

### Propensity score matching

Propensity score matching was performed using a logistic regression, including variables such as age, sex, diabetes severity, antidiabetic medications, concomitant medications, HbA1c and eGFR (Table S2). These variables were selected based on their potential to influence both treatment assignment and outcomes, as identified from previous literature and clinical expertise. A 1:1 propensity score matching was performed. Satisfying a standardized difference of less than 0.1 in all variables indicated a balanced cohort.

### Outcomes

The primary outcomes were changes in laboratory parameters related to kidney function, including eGFR, blood pressure, hematocrit, electrolytes, and serum uric acid.

The secondary outcomes were adverse renal events including AKI, albuminuria or proteinuria, end-stage kidney disease, eGFR declines by ≥ 30% from baseline, a composite of these kidney adverse outcomes, weight loss, increased urination, and reduction of antihypertensive medications. For the secondary categorical outcomes, operational definitions were used in this study (Table S3).

### Statistical analysis

We employed a linear mixed model for continuous outcomes to account for the correlation between repeated measurements within individuals and to handle unbalanced data. Linear mixed-effects models were calculated, setting fixed effects for diabetes drugs, duration of drug use in days, and the interaction between diabetes drugs and duration of drug use. A random intercept was calculated for the patient factor. The results were presented as β estimates and 95% confidence intervals (CIs). Categorical outcomes were analyzed using the Cox proportional hazard model, and each result was presented as hazard ratios (HRs) and 95% CIs.

The main analysis in this study was performed using a modified intention-to-treat analysis. The follow-up continued until the occurrence of the study outcome, the end of study data (date of transfer out, date of death, or date of study end), the date on which a SGLT2 inhibitor and a DPP4 inhibitor were prescribed in combination, or the date on which the index drug was switched to another antidiabetic drug. The follow-up period was up to 13 months, which is longer than the approximately 12 months typically required for recovery from the acute dip in eGFR associated with SGLT2 inhibitors[15]. Sensitivity analyses were performed using an as-treated analysis, in which the follow-up was additionally censored at the end date of the first continuous treatment of the index drug plus a grace period (30 days after the end of the last prescriptions supply) (Figure S1). No imputation was performed for outcome variables during follow-up, and for baseline variables, the most recent value prior to the index date was used. All analyses were performed using SAS 9.4 version (SAS Institute Inc., Cary, NC, USA).

## Ethics approval

This study was approved by the Institutional Review Board of Seoul National University Hospital (IRB No. 2106–016-1224, approved on 6 July 2021) and adhered to the Declaration of Helsinki. The requirement for informed consent was waived by the Institutional Review Board because all personally identifiable information had been removed in compliance with relevant guidelines and regulations.

## Results

### Baseline characteristics of study population

**Fig. 2:**
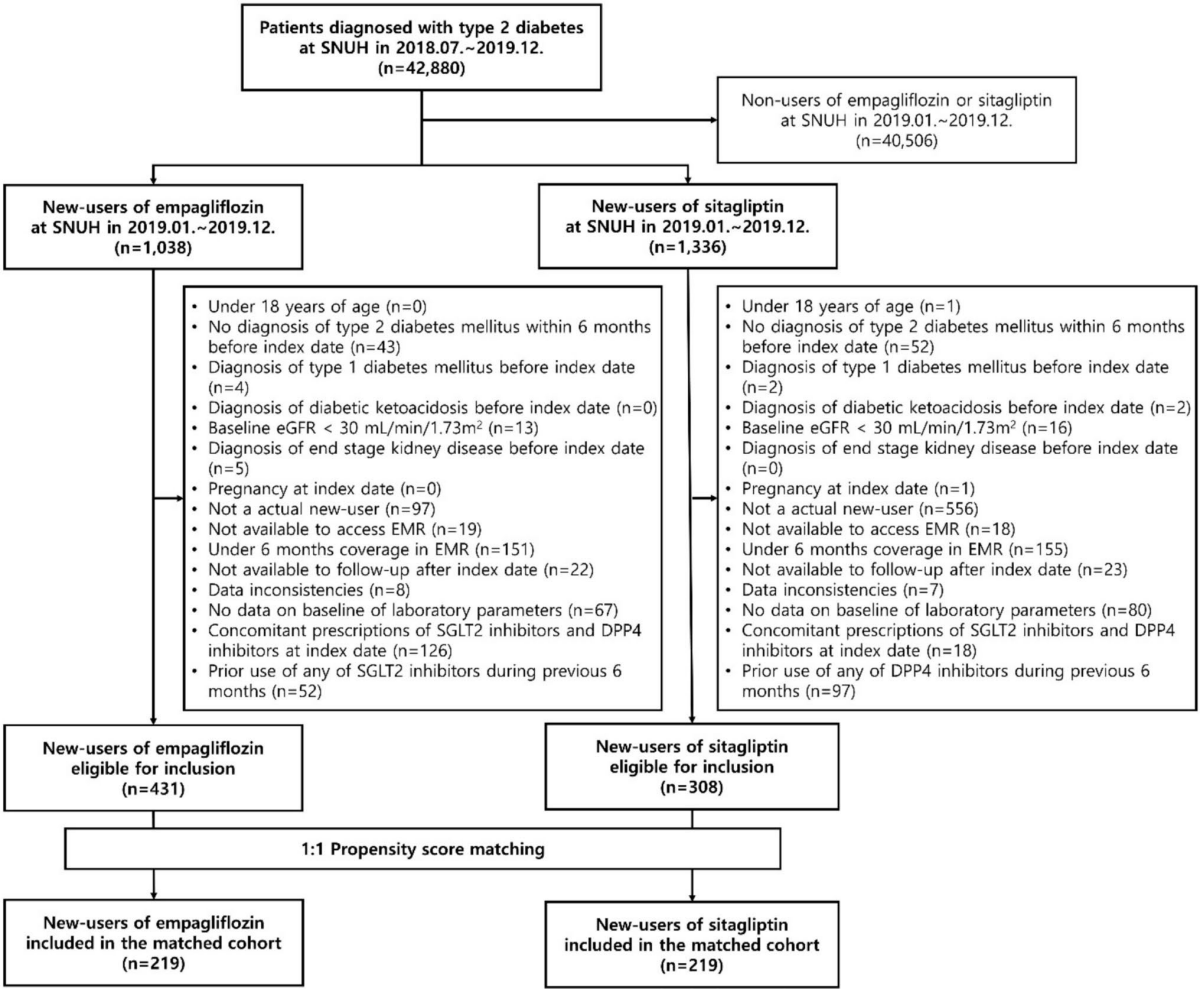
Flow chart of the study. eGFR, estimated glomerular filtration rate; SNUH, Seoul National University Hospital; EMR, electronic medical records; SGLT2, sodium-glucose cotransporter 2; DPP4, dipeptidyl peptidase-4

From July 2018 to December 2019, 42,880 patients diagnosed with T2D were identified (Fig. 2). Among 1,038 new-users of empagliflozin and 1,336 new-users of sitagliptin, 431 and 308 patients, respectively, satisfied the inclusion and exclusion criteria. After 1:1 propensity score matching, a cohort of 219 patients in each group was constructed. Baseline characteristics for the cohort before and after matching were presented in Table 2. After 1:1 propensity score matching between new-users of empagliflozin and sitagliptin, the baseline characteristics were similar in both groups (standardized difference < 0.1, for all variables).

**Table 2:** Baseline characteristics before and after propensity score matching

Characteristics	Before matching			After matching		
	Empagliflozin (n = 431)	Sitagliptin (n = 308)	Std diff	Empagliflozin (n = 219)	Sitagliptin (n = 219)	Std diff
*Demographic characteristics*						
Age (years)^a^	63.1 ± 12.1	64.8 ± 12.8	− 0.13	63.8 ± 11.6	64.3 ± 13.1	− 0.05
Male sex, no. (%)	278 (64.5)	165 (53.6)	0.22	121 (55.3)	119 (54.3)	0.02
Diabetes severity, no. (%)						
Diabetic retinopathy	80 (18.6)	50 (16.2)	0.06	34 (15.5)	39 (17.8)	− 0.06
Diabetic neuropathy	28 (6.5)	27 (8.8)	− 0.09	18 (8.2)	18 (8.2)	0.00
Diabetic nephropathy	44 (10.2)	26 (8.4)	0.06	21 (9.6)	22 (10.1)	− 0.02
Peripheral vascular disease	8 (1.9)	6 (2.0)	− 0.01	3 (1.4)	4 (1.8)	− 0.04
CKD stages 3–5	10 (2.3)	2 (0.7)	0.14	3 (1.4)	2 (0.9)	0.04
Amputations	2 (0.5)	0	0.10	0	0	0
*Antidiabetic comedications, no. (%)*						
Metformin	387 (89.8)	282 (91.6)	− 0.06	196 (89.5)	196 (89.5)	0.00
Sulfonylureas	159 (36.9)	76 (24.7)	0.27	67 (30.6)	64 (29.2)	0.03
Thiazolidinediones	9 (2.1)	6 (2.0)	0.01	6 (2.7)	6 (2.7)	0.00
Meglitinides	0	0	0	0	0	0
α-glucosidase inhibitors	0	1 (0.3)	− 0.08	0	0	0
GLP-1 receptor agonists	4 (0.9)	1 (0.3)	0.08	1 (0.5)	1 (0.5)	0.00
Insulins	61 (14.2)	34 (11.0)	0.09	23 (10.5)	26 (11.9)	− 0.04
*Concomitant medications, no. (%)*						
ACEIs/ARBs	232 (53.8)	111 (36.0)	0.36	92 (42.0)	91 (41.6)	0.01
CCBs	191 (44.3)	76 (24.7)	0.42	66 (30.1)	69 (31.5)	− 0.03
Diuretics	85 (19.7)	37 (12.0)	0.21	29 (13.2)	33 (15.1)	− 0.05
Statins	336 (78.0)	200 (64.9)	0.29	154 (70.3)	161 (73.5)	− 0.07
*Baseline of clinical laboratory parameters*^*a*^						
HbA1c (%)	7.7 ± 1.2	8.0 ± 1.6	− 0.21	7.8 ± 1.3	7.7 ± 1.3	0.04
eGFR (mL/min/1.73m^2^)	79.7 ± 18.3	80.3 ± 19.0	− 0.03	80.2 ± 18.6	80.1 ± 19.6	0.00
Sodium (mmol/L)	140.2 ± 2.2	139.9 ± 2.5	0.12	140.1 ± 2.3	140.1 ± 2.4	0.02
Potassium (mmol/L)	4.5 ± 0.4	4.4 ± 0.4	0.19	4.4 ± 0.4	4.4 ± 0.4	0.02
Chloride (mmol/L)	103.2 ± 2.8	103.1 ± 3.0	0.05	103.3 ± 2.8	103.2 ± 3.0	0.06
Calcium (mg/dL)	9.4 ± 0.4	9.3 ± 0.4	0.13	9.3 ± 0.4	9.3 ± 0.4	0.05
Phosphorus (mg/dL)	3.4 ± 0.5	3.4 ± 0.5	− 0.08	3.4 ± 0.6	3.4 ± 0.5	− 0.01
Uric acid (mg/dL)	5.1 ± 1.4	4.7 ± 1.4	0.30	4.9 ± 1.3	4.9 ± 1.4	0.02
Hemoglobin (g/dL)	13.9 ± 1.6	13.5 ± 1.8	0.26	13.7 ± 1.6	13.7 ± 1.5	− 0.04
Hematocrit (%)	41.7 ± 4.4	40.4 ± 4.8	0.26	40.9 ± 4.3	41.1 ± 4.1	− 0.06
Platelet (× 10^3^/μL)	236.4 ± 62.6	239.1 ± 75.3	− 0.04	237.6 ± 64.9	236.9 ± 73.3	0.01
WBC (× 10^3^/μL)	7.3 ± 1.9	6.8 ± 2.3	0.24	6.9 ± 1.6	6.9 ± 2.2	0.02
RBC (× 10^6^/μL)	4.6 ± 0.6	4.5 ± 0.6	0.25	4.5 ± 0.5	4.5 ± 0.5	− 0.01
Systolic BP (mmHg)	134.0 ± 16.5	131.3 ± 17.3	0.16	132.4 ± 16.8	132.2 ± 16.8	0.01
Diastolic BP (mmHg)	77.5 ± 11.4	76.8 ± 12.4	0.06	77.0 ± 11.3	77.2 ± 12.0	− 0.02
Pulse rate (beats/min)	81.0 ± 12.7	80.9 ± 14.1	0.01	80.8 ± 13.1	80.5 ± 13.4	0.03

Std.diff, standardized difference; CKD, chronic kidney disease; GLP-1, glucagon-like peptide-1; ACEIs, angiotensin-converting enzyme inhibitors; ARBs, angiotensin receptor blockers; CCBs, calcium-channel blockers; eGFR, estimated glomerular filtration rate; WBC, white blood cell; RBC, red blood cell; BP, blood pressure

^a^Data are presented as mean ± standard deviation

### Effect on renal function

Empagliflozin showed significant changes in the laboratory parameters related to kidney function according to the duration of drug use compared to sitagliptin (Table 3). Empagliflozin was associated with a significantly slower decline in eGFR compared to sitagliptin, depending on the duration of drug use in days (β estimates 0.00013; 95% CI, 0.00003–0.00023; *p* < 0.05) suggesting a potential renoprotective effect. However, the trends in the changes of serum phosphorus and serum uric acid were significantly different between empagliflozin and sitagliptin depending on the duration of drug use. Compared to sitagliptin, the rate of change in serum phosphorus increased from baseline in the empagliflozin group as the duration of drug use extended. However, the rate of change in serum uric acid decreased from baseline in the empagliflozin group. Only diastolic blood pressure was significantly decreased in empagliflozin compared to sitagliptin, independent of the duration of drug use.

**Table 3:** Linear mixed model’s β estimates for laboratory parameters related to kidney function

Laboratory parameters^a^	Drug^b^		Duration of drug use in days		Drug*Duration of drug use in days	
	β estimate (× 10^–3^)	95% CI (× 10^–3^)	β estimate (× 10^–3^)	95% CI (× 10^–3^)	β estimate (× 10^–3^)	95% CI (× 10^–3^)
eGFR (mL/min/1.73m^2^)	8.82	− 17.17 to 34.81	**− 0.11**	**− 0.18 to − 0.04**	**0.13**	**0.03 to 0.23**
Systolic BP (mmHg)	− 2.74	− 24.03 to 18.55	− 0.02	− 0.09 to 0.04	− 0.04	− 0.13 to 0.05
Diastolic BP (mmHg)	**− 25.81**	**− 50.42 to − 1.21**	**− 0.12**	**− 0.19 to − 0.04**	0.06	− 0.04 to 0.16
Hematocrit (%)	− 4.58	− 25.34 to 16.17	− 0.06	− 9.86 to 17.97	**0.20**	**0.11 to 0.29**
Sodium (mmol/L)	− 2.17	− 5.56 to 1.22	**0.22**	**0.002 to 0.03**	− 0.006	− 0.02 to 0.01
Potassium (mmol/L)	0.42	− 16.72 to 17.56	**− 0.10**	**− 0.17 to − 0.03**	0.05	− 0.04 to 0.14
Chloride (mmol/L)	− 0.72	− 5.70 to 4.27	**0.02**	**0.003 to 0.04**	− 0.02	− 0.05 to 0.002
Calcium (mg/dL)	6.28	− 1.94 to 14.50	0.02	− 0.009 to 0.04	− 0.02	− 0.05 to 0.02
Phosphorus (mg/dL)	4.52	− 20.61 to 29.65	**0.10**	**0.02 to 0.17**	**0.12**	**0.01 to 0.23**
Uric acid (mg/dL)	10.53	− 30.21 to 51.27	**0.19**	**0.09 to 0.30**	**− 0.30**	**− 0.44 to − 0.15**

CI, confidence interval; eGFR, estimated glomerular filtration rate; BP, blood pressure

^a^Data were analyzed as log-transformed ratios of the change from baseline.

^b^The effect was estimated as empagliflozin against sitagliptin

Bold font indicates statistical significance (*p* < 0.05)

### Risk of kidney-related adverse events

The risk of composite kidney adverse outcomes was not significantly different between the empagliflozin and sitagliptin groups. When assessing the risk of each renal adverse event included in the composite kidney outcomes, decreasing trends were observed in AKI, albuminuria or proteinuria, and eGFR declines of ≥ 30% from baseline, although these were not statistically significant (Table 4). No cases of end-stage kidney disease were identified in either group. An increased risk of weight loss (HR 2.95; 95% CI, 2.01–4.33) and increased urination (HR 4.05; 95% CI, 1.14–14.34) was observed in the empagliflozin group. The reduction in antihypertensive medications was not significantly different.

**Table 4:** Association of kidney-related adverse outcomes in the matched population

Outcomes	Empagliflozin (n = 219)			Sitagliptin (n = 219)		Hazard ratio (95% CI)
	Events (%)	Events/1,000 patient years		Events (%)	Events/1,000 patient years	
Composite kidney outcomes^a^	34 (15.5)	184.8		43 (19.6)	237.6	0.78 (0.50–1.22)
Acute kidney injury	2 (0.9)	10.0		7 (3.2)	35.2	0.29 (0.06–1.37)
Albuminuria/Proteinuria	27 (12.3)	142.9		34 (15.5)	183.8	0.78 (0.47–1.30)
End-stage kidney disease	0	NA		0	NA	NA
eGFR decline ≥ 30% from baseline	7 (3.2)	35.5		11 (5.0)	55.8	0.64 (0.25–1.65)
Weight loss	89 (40.6)	589.4		37 (16.9)	198.9	**2.95 (2.01–4.33)**
Increased urination	12 (5.5)	60.9		3 (1.4)	14.9	**4.05 (1.14–14.34)**
Reduction of antihypertensive medications	2 (0.9)	10.0		2 (0.9)	10.0	0.99 (0.14–7.03)

CI, confidence interval; eGFR, estimated glomerular filtration rate; NA, not available

^a^Acute kidney injury, albuminuria/proteinuria, end-stage kidney disease, eGFR decline ≥ 30% from baseline

Bold font indicates statistical significance (*p* < 0.05)

### Sensitivity analyses

In the sensitivity analysis using the as-treated method, the results of the linear mixed models showed that the results of the main analyses were robust, except for diastolic blood pressure, which became non-significant, unlike in the main analysis. Additionally, an increase in hematocrit was newly and significantly observed in the empagliflozin group (Table S4). The results of the sensitivity analyses for secondary outcomes were consistent with the main analysis (Table S5).

## Discussion

Our findings of slower eGFR decline with empagliflozin use are consistent with previous randomized controlled trials, but extend these results to a broader T2D population. The observed effect size suggests that over a year, patients on empagliflozin might experience approximately 4.9% less eGFR decline compared to those on sitagliptin. While this difference may seem small, it could translate to significant long-term renal benefits, potentially delaying the progression to end-stage kidney disease.

This study emulated a target trial using EMR data to compare renal effects of empagliflozin compared to sitagliptin, minimizing biases common in observational designs. This study evaluated not only the risk of adverse events occurrence, but also the exact laboratory changes depending on the duration of drug use using real-world data to generalize the results of RCTs. We found that there was no significant increase in adverse kidney outcomes in empagliflozin users compared to sitagliptin users, and empagliflozin exhibited significant indirect kidney protective effects, such as weight loss, increased urination, and decreased diastolic blood pressure. Additionally, eGFR changes improved with empagliflozin compared to sitagliptin with extended use.

These findings suggest that empagliflozin may offer cumulative renal benefits with long-term use. The renoprotective effect observed in this study is consistent with the results of previous RCTs on empagliflozin conducted with patients having cardiovascular or renal diseases[4, 20–23]. In this study, about 90% of the patients in the matched cohort were using the drug in combination with metformin. The renoprotective effect of empagliflozin can be explained by several mechanisms such as natriuresis and osmotic diuresis[24, 25]. Additionally, a decrease in sodium reabsorption in the proximal tubule reduces oxidative stress, hypoxia-induced fibrosis is suppressed, and production of inflammatory substances is reduced[25, 26]. Since this study was a single-institutional study conducted immediately after empagliflozin’s approval in South Korea, recruiting a sufficient number of patients was challenging. Therefore, we were unable to confirm the direct renoprotective effect through statistically significant reduction in risk of the composite kidney outcomes in empagliflozin compared to sitagliptin. To achieve maximum statistical significance in this study, we utilized detailed EMR data including laboratory test data and found reduced HR in risk of the composite kidney outcomes. Further studies, such as multicenter research, will allow for clearer significance for the composite kidney outcomes.

The serum phosphorus levels showed a significant increase with longer use in empagliflozin compared to sitagliptin. A secondary analysis of an RCT comparing empagliflozin with placebo observed transient increase in phosphate after 3 days[27]. Our finding supports the previous study with a larger population and longer follow-up period, and we additionally found that increased phosphorus does not fully recover with long term use of empagliflozin. Serum phosphate competes with glucose for sodium-dependent renal reabsorption, and inhibiting SGLT2 increases the reabsorption of phosphate[28]. Based on this mechanism, it has been suggested that, as a class effect, the increase in serum phosphorus stimulates the FGF23/1, 25-dihydroxyvitamin D/PTH axis when SGLT2 is inhibited[29]. Since elevated serum phosphorus is associated with fractures and renal complications, careful observation is required when using empagliflozin. Although the increase of serum phosphorus in this study was not large but significant depending on the duration of drug use, routine monitoring of serum phosphorus could be considered especially in patients who take empagliflozin for a long period of time, longer than 13 months, which is the follow-up period of this study.

The increase in serum uric acid tended to gradually decrease in the empagliflozin group compared to the sitagliptin group. This is consistent with the results of decreased serum uric acid levels observed in the EMPA-REG OUTCOME trial after taking empagliflozin[30], supporting the renoprotective effect of empagliflozin.

The diastolic blood pressure was significantly lower in empagliflozin group compared to sitagliptin group; however, this significance was not robust in the sensitivity analysis. The β estimate for the drug’s effect on diastolic blood pressure was offset by a wide 95% CI, indicating significant patient variability that requires a larger sample size for statistical significance; however, the as-treated sensitivity analysis reduced the sample size by excluding periods of poor compliance. Therefore, the effect of empagliflozin on diastolic blood pressure may be more clearly confirmed through additional studies with larger sample sizes. Additionally, both the risk of weight loss and the risk of increased urination were significantly increased in empagliflozin users in this study. These findings are consistent with previous studies[31, 32]. The initial weight loss from empagliflozin is due to dehydration and caloric reduction through urinary glucose excretion[33]. However, in the long term, it can be explained as a result of increased fatty acid oxidation and free fatty acid excretion from adipocytes[34]. The insignificant change in hematocrit supports that the weight loss is a result of a decrease in body fat rather than a decrease in body fluid. Similarly, contrary to the expectation that osmotic diuresis will lead to loss of body volume, a clinical trial suggests that it can maintain body volume by inducing vasopressin-induced reabsorption[35]. These studies suggest that taking empagliflozin increases the risk of increased urination and weight loss; however, it leads to fat loss without significant loss of body fluids. These benefits may contribute to obesity reduction, aiding long-term metabolic and renal risk management in T2D.

This study introduced the first application of TTE method in Korea to assess the effects of empagliflozin on renal function by reviewing EMR in Korea. Emulating each component of the pre-built protocol of the hypothetical randomized trial makes it possible to minimize the bias of retrospective observational studies and interpret the results similar to an RCT. Despite the inclusion of all patients who took empagliflozin, regardless of the presence or absence of specific underlying diseases, which helps to generalize the results, we set an appropriate comparator group and adjusted for possible confounders. This method can be broadly used to address other research questions with other real-world data. Additionally, by analyzing laboratory test data using the linear mixed model, the various intervals and number of measurements of laboratory parameters in the actual clinical environment were all reflected in the analysis.

This study has several limitations. First, it is a single-institution study using the EMR of a tertiary hospital. Therefore, prescriptions from other hospitals could not be collected, making it difficult to detect new-user. However, we choose this database to evaluate accurate changes in renal function, and this limitation is inevitable. To address this, we set a six-month prescreening period to cover drug refill gaps and excluded patients without a diagnosis of T2D within six months before the index date, as these patients could have received antidiabetic drugs elsewhere. Second, the possibility of unmeasured confounding factors and selection bias cannot be excluded. However, these were minimized using propensity score matching and active controls. Additionally, all the medical records within the follow-up period were reviewed to minimize the missing detection of adverse events. Third, the follow-up period was shorter than previous RCTs which first observed renoprotective effect of empagliflozin[4]. However, the primary outcome of this RCT was cardiovascular risk, which requires long-term follow-up. The acute dip in eGFR with SGLT2 inhibitors is usually recovered in about 12 months, showing a protective effect[15]. Therefore, our follow-up period of 13 months can be long enough for detecting the renal effectiveness of empagliflozin. Fourth, this study was conducted exclusively with Korean patients, which may limit generalizability of the findings to other populations. While our study focused on Korean patients, several aspects of our findings suggest applicability to other populations. The mechanisms underlying the renoprotective effects of SGLT2 inhibitors are likely consistent across different ethnic groups. Moreover, our use of TTE methodology enhances the internal validity of our results, potentially increasing their generalizability to real-world settings in other countries. An available literature on empagliflozin pharmacokinetics in different ethnic groups suggests minimal clinically significant differences[36], further supporting the potential extrapolation of our findings to other populations. The mechanistic insights, such as the reduction in serum uric acid levels, are likely consistent across populations[37], supporting the broader applicability of our findings. Fifth, this study utilized a retrospective cohort based on EMR data available between July 2018 and December 2019, which limited the ability to perform a priori sample size calculation. Given the low incidence of composite renal outcomes observed, our study may have been underpowered to detect statistically significant differences in these endpoints. Future studies with larger sample sizes and longer follow-up periods are needed to confirm these effects.

## Conclusion

Our findings, alongside studies from other populations, contribute to a more comprehensive understanding of the renal effects of empagliflozin in diverse T2D patients. The renoprotective effects observed, as demonstrated in comparison to sitagliptin, suggest empagliflozin may be preferable option for T2D patients at renal risk, though serum phosphorus should be carefully monitored.

## Supplementary Information

Below is the link to the electronic supplementary material.

Supplementary file1 (DOCX 80 KB)

## Data Availability

The data presented in this study are available upon request from the corresponding author.
