# Supplementary material for: The effect of empagliflozin on renal outcomes compared to sitagliptin with type 2 diabetes: target trial emulation using electronic medical records
Source: Int J Clin Pharm. 2025 Oct 23;48(2):569–79. doi: 10.1007/s11096-025-02024-9 (PMC12992483; doi:10.1007/s11096-025-02024-9)
Supplement: Supplementary file 1 — Supplementary file1 (DOCX 80 KB) [file 11096_2025_2024_MOESM1_ESM.docx]

**The effect of empagliflozin on renal function compared to sitagliptin with type 2 diabetes: Target trial emulation using electronic medical records**

**Author names**

Min Ju Kang^1+^, Hyun Kyung Lee^1+^, Minoh Ko^1,2^, Ha Young Jang^1,3^, In-Wha Kim^4^, Jung Mi Oh^1,4,5*^

^+^ These authors contributed equally to this work.

**Supplementary Appendix**

# Table S1. The lists of marketed drugs of SGLT2 inhibitors and DPP4 inhibitors in Korea

| **Drug class** | **Generic name** | **ATC code** |
| --- | --- | --- |
| SGLT2i | Dapagliflozin  Empagliflozin  Ertugliflozin  Ipragliflozin | A10BK01  A10BK03  A10BK04  A10BK05 |
| DPP4i | Alogliptin  Anagliptin  Evogliptin  Gemigliptin  Linagliptin  Saxagliptin  Sitagliptin  Teneligliptin  Vildagliptin | A10BH04  A10BH  A10BH07  A10BH06  A10BH05  A10BH03  A10BH01  A10BH08  A10BH02 |
| Combination drugs containing SGLT2i or DPP4i | Metformin and anagliptin  Metformin and sitagliptin  Metformin and vildagliptin  Pioglitazone and alogliptin  Metformin and saxagliptin  Metformin and linagliptin  Metformin and alogliptin  Metformin and dapagliflozin  Metformin and gemigliptin  Metformin and empagliflozin  Metformin and evogliptin  Metformin and teneligliptin  Gemigliptin and rosuvastatin | A10BD  A10BD07  A10BD08  A10BD09  A10BD10  A10BD11  A10BD13  A10BD15  A10BD18  A10BD20  A10BD22  A10BD28  A10BH52 |

Abbreviations: ATC, anatomical therapeutic chemical; SGLT2i, Sodium-glucose cotransporter 2 inhibitors; DPP4i, Dipeptidyl peptidase-4 inhibitors

# Table S2. Selected variables used in propensity score matching

| **Category** | **Variables** |
| --- | --- |
| Diabetic severity | Diabetic retinopathy  Diabetic neuropathy  Diabetic nephropathy  Peripheral vascular disease  Chronic kidney disease stages 3-5  Amputations |
| Antidiabetic comedications | Metformin  Sulfonylureas  Thiazolidinediones  Meglitinides  α-glucosidase inhibitors  GLP-1 receptor agonists  Insulins |
| Concomitant medications | Angiotensin-converting enzyme inhibitors/Angiotensin Ⅱ receptor blockers  Calcium-channel blockers  Diuretics  Statins |

Abbreviations: GLP-1, glucagon-like peptide-1.

# Table S3. Definitions of categorical outcomes used in the study

| **Outcomes** | **Definitions** |
| --- | --- |
| Composite kidney outcomes | Any one of the following outcomes: 1) Acute kidney injury 2) Albuminuria/Proteinuria 3) End stage kidney disease 4) Estimated glomerular filtration rate declines of by ≥ 30% from baseline |
| Acute kidney injury (AKI) | Any one of the following criteria: 1) Diagnostic codes: identified new diagnosis of ICD-10 code N17 2) Clinical notes: identified newly recorded terms indicating acute kidney injury after the index date 3) Laboratory parameters: increase in serum creatinine of by ≥ 0.3 mg/dL within 48 hours; or increase in serum creatinine to ≥ 1.5 times baseline; or urine volume < 0.5 mL/kg/h for 6 hours |
| Albuminuria/Proteinuria | Any one of the following criteria: 1) Diagnostic codes: identified new diagnosis of ICD-10 codes R80, E11.2, E12.2, E13.2, E14.2, N08.3 2) Clinical notes: identified newly recorded terms indicating albuminuria or proteinuria 3) Laboratory parameters for proteinuria: identified new-onset proteinuria defined as urine protein-to-creatinine ratio(PCR) ≥ 50 mg/g or protein excretion rate (PER) ≥ 150 mg/24hr 4) Laboratory parameters for albuminuria: identified new-onset albuminuria defined as urine albumin-to-creatinine rate (ACR) ≥ 30 mg/g or albumin excretion rate (AER) ≥ 30mg/24hr or urine albumin dipstick of ≥ trace |
| End stage kidney disease | Any one of the following criteria: 1) Diagnostic codes: identified new diagnosis of ICD-10 codes N18.5, Y84.1 2) Clinical notes: Identified newly recorded terms indicating end stage kidney disease, dialysis for 30 days or more, or kidney transplantation 3) Laboratory parameters: a decline in estimated glomerular filtration rate of ≥ 30% from baseline |
| Estimated glomerular filtration rate declines of by ≥ 30% from baseline | Laboratory parameters: a decline in estimated glomerular filtration rate ≥ 30% from baseline |
| Weight loss | Any one of the following criteria: 1) Clinical notes: identified newly recorded terms indicating weight loss or newly recorded weight loss of > 3% of the baseline body weight 2) Laboratory parameters: a loss of > 5% of the body weight over a period of 6-12 months |
| Increased urination | Any one of the following criteria: 1) Diagnostic codes: identified new diagnosis of ICD-10 code R35 2) Clinical notes: identified newly recorded terms indicating frequent urination, polyuria, nocturia |
| Reduction of antihypertensive medications | Medications: reduction of any antihypertensive medications based on total daily dose, potency, and the number of active ingredients |

# Table S4. The results of sensitivity analysis on the change of laboratory parameters in the matched population

| **Laboratory parameters^†^** | **Drug^‡^** | |  | **Duration of drug use** | |  | **Drug*Duration of drug use** | |
| --- | --- | --- | --- | --- | --- | --- | --- | --- |
|  | **β estimate**  **(x10^-3^)** | **95% CI**  **(x10^-3^)** |  | **β estimate**  **(x10^-3^)** | **95% CI**  **(x10^-3^)** |  | **β estimate**  **(x10^-3^)** | **95% CI**  **(x10^-3^)** |
| eGFR (mL/min/1.73m^2^) | 8.06 | -17.78 – 33.91 |  | **-0.13** | **-0.20 – -0.06** |  | **0.15** | **0.04 – 0.25** |
| Systolic BP (mmHg) | 0.15 | -20.33 – 20.62 |  | -0.02 | -0.08 – 0.05 |  | -0.05 | -0.14 – 0.04 |
| Diastolic BP (mmHg) | -21.47 | -45.00 – 2.06 |  | **-0.11** | **-0.18 – -0.03** |  | 0.05 | -0.05 – 0.15 |
| Hematocrit (%) | -3.34 | -23.70 – 17.03 |  | **-0.08** | **-0.14 – -0.01** |  | **0.26** | **0.17 – 0.36** |
| Sodium (mmol/L) | -2.41 | -5.79 – 0.96 |  | 0.01 | -0.002 – 0.03 |  | -0.00002 | -0.02 – 0.02 |
| Potassium (mmol/L) | 0.16 | -17.03 – 17.35 |  | **-0.11** | **-0.18 – -0.03** |  | 0.08 | -0.02 – 0.17 |
| Chloride (mmol/L) | -1.00 | -5.95 – 3.94 |  | 0.02 | -0.0004 – 0.04 |  | -0.02 | -0.05 – 0.003 |
| Calcium (mg/dL) | 7.11 | -0.96 – 15.17 |  | 0.01 | -0.01 – 0.04 |  | -0.006 | -0.04 – 0.03 |
| Phosphorus (mg/dL) | 4.19 | -20.77 – 29.14 |  | **0.10** | **0.02 – 0.18** |  | **0.16** | **0.05 – 0.27** |
| Uric acid (mg/dL) | 12.63 | -27.94 – 53.19 |  | **0.20** | **0.09 – 0.31** |  | **-0.30** | **-0.44 – -0.14** |

Abbreviations: CI, confidence interval; eGFR, estimated glomerular filtration rate; BP, blood pressure.

†Data were analyzed as log-transformed ratios of the change from baseline.

‡The effect was estimated as empagliflozin against sitagliptin.

Bold font indicates statistical significance (*p*<0.05).

# Table S5. The results of sensitivity analysis on kidney-related adverse outcomes in the matched population

| **Outcomes** | **Empagliflozin**  **(n=219)** | |  | **Sitagliptin**  **(n=219)** | | **Hazard ratio**  **(95% CI)** |
| --- | --- | --- | --- | --- | --- | --- |
|  | **Events (%)** | **Events/1,000 patient years** |  | **Events (%)** | **Events/1,000 patient years** |  |
| Composite kidney outcomes^a^ | 34 (15.5) | 196.5 |  | 43 (19.6) | 251.5 | 0.78 (0.50 – 1.23) |
| Acute kidney injury | 2 (0.9) | 10.7 |  | 7 (3.2) | 37.2 | 0.29 (0.06 – 1.38) |
| Albuminuria/Proteinuria | 26 (11.9) | 147.7 |  | 32 (14.6) | 182.9 | 0.81 (0.49 – 1.37) |
| eGFR decline ≥ 30% from baseline | 7 (3.2) | 37.8 |  | 11 (5.0) | 59.1 | 0.64 (0.25 – 1.66) |
| Weight loss | 84 (38.4) | 583.3 |  | 35 (16.0) | 198.9 | **2.92 (1.96 – 4.33)** |
| Increased urination | 11 (5.0) | 60.9 |  | 3 (1.4) | 15.9 | **3.74 (1.04 – 13.39)** |
| Reduction of antihypertensive medications | 2 (0.9) | 10.7 |  | 2 (0.9) | 10.6 | 1.01 (0.14 – 7.14) |

Abbreviations: CI, confidence interval; eGFR, estimated glomerular filtration rate.

^a^acute kidney injury, albuminuria/proteinuria, end stage kidney disease, eGFR decline ≥ 30% from baseline

| **Case 1. Exposure period with prescription gaps ≤ 30 days**  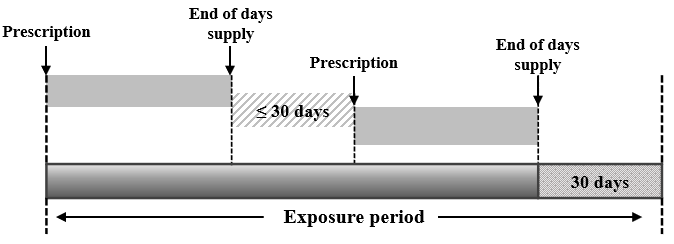  **Case 2. Exposure period with prescription gaps > 30 days**  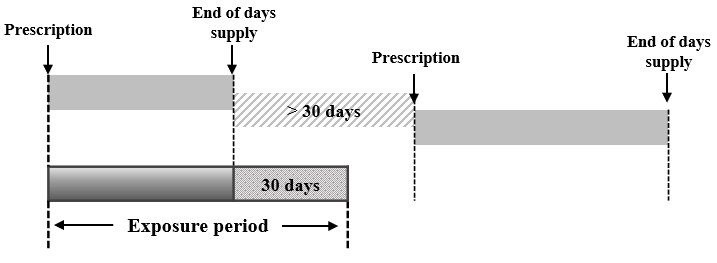  **Case 3. Exposure period with overlapping prescriptions**  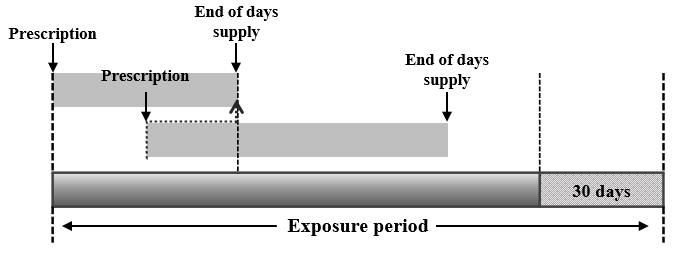 |
| --- |

# Figure S1. Definitions of exposure period using an as-treated method
